# Incremental 3D Cuboid Modeling with Drift Compensation

**DOI:** 10.3390/s19010178

**Published:** 2019-01-06

**Authors:** Masashi Mishima, Hideaki Uchiyama, Diego Thomas, Rin-ichiro Taniguchi, Rafael Roberto, João Paulo Lima, Veronica Teichrieb

**Affiliations:** 1 Graduate School of Information Science and Electrical Engineering, Kyushu University, Fukuoka 819-0395, Japan; 2 Library, Kyushu University, Fukuoka 819-0395, Japan; uchiyama@limu.ait.kyushu-u.ac.jp; 3 Faculty of Information Science and Electrical Engineering, Kyushu University, Fukuoka 819-0395, Japan; diego_thomas@limu.ait.kyushu-u.ac.jp (D.T.); rin@kyudai.jp (R.-i.T.); 4 Voxar Labs, Centro de Informática, Universidade Federal de Pernambuco, Recife 50740-560, Brazil; rar3@cin.ufpe.br (R.R.); jpsml@cin.ufpe.br (J.P.L.); vt@cin.ufpe.br (V.T.); 5 Departamento de Computação, Universidade Federal Rural de Pernambuco, Recife 52171-900, Brazil

**Keywords:** geometric shape, cuboid, incrementally structural modeling, point cloud

## Abstract

This paper presents a framework of incremental 3D cuboid modeling by using the mapping results of an RGB-D camera based simultaneous localization and mapping (SLAM) system. This framework is useful in accurately creating cuboid CAD models from a point cloud in an online manner. While performing the RGB-D SLAM, planes are incrementally reconstructed from a point cloud in each frame to create a plane map. Then, cuboids are detected in the plane map by analyzing the positional relationships between the planes, such as orthogonality, convexity, and proximity. Finally, the position, pose, and size of a cuboid are determined by computing the intersection of three perpendicular planes. To suppress the false detection of the cuboids, the cuboid shapes are incrementally updated with sequential measurements to check the uncertainty of the cuboids. In addition, the drift error of the SLAM is compensated by the registration of the cuboids. As an application of our framework, an augmented reality-based interactive cuboid modeling system was developed. In the evaluation at cluttered environments, the precision and recall of the cuboid detection were investigated, compared with a batch-based cuboid detection method, so that the advantages of our proposed method were clarified.

## 1. Introduction

Owing to the advance of visual odometry and simultaneous localization and mapping (SLAM), the automated control of cars, drones, and robots has been achieved by generating a point cloud-based 3D map. Although localization can be performed by using the map, the map is simply composed of sets of a point, and does not represent semantics in the environment. Toward 3D-scene understanding, it is important to convert a point cloud into an object-level representation as the high-level knowledge of the scene geometry. Planes, cylinders, and spheres are an example of a low-level parametric primitive representation to compose the scene geometry. The recognition of such primitive shapes is an important task for various applications such as obstacle avoidance and object grasping [1].

A cuboid, which is the target shape for the CAD conversion in this paper, is also considered as an informative shape representation because there exist many types of cuboids in our environment. For instance, delivery boxes used in logistics and product packages in markets are practical examples. To achieve automated robot manipulation in such environments, techniques to recognize cuboids under cluttered conditions are often required. In the literature of this field, cuboid detection has been performed by using a monocular RGB image [2,3,4,5,6] or a point cloud generated with a single RGB-D image [7,8,9,10,11,12,13,14]. For outdoor environments, the approaches for generating cuboid-based building models have been proposed according to the density of a point cloud with Light Detection and Ranging (LIDAR) [15,16,17]. Generally, these methods are based on offline batch processing such that recognition is only performed with a single observation. Typically, they suffer from both false positives and false negatives due to the noisy 3D map. To solve the problem, we investigated a novel sequential approach to suppress false detection by incorporating multiple observations with temporal filtering in an online manner [18,19].

In this paper, we propose a framework of incremental cuboid modeling by using the mapping results of an RGB-D SLAM system. In our framework, an RGB-D SLAM system was used as an external tracker to incrementally produce a point cloud of the environment so that any RGB-D SLAM system could be applied, as similar to the framework in Reference [20]. We focus on stably modeling cuboids from the incrementally updated point cloud. At every frame, planes are incrementally reconstructed and updated from a point cloud-based map [21], and are used as the input to our framework. Then, planes are clustered to compose cuboids by analyzing three plane-positional relationships, namely, orthogonality, convexity, and proximity. To accurately reconstruct a cuboid, a cluster of three perpendicular planes is first selected, and their intersection is computed [22]. By determining three perpendicular cuboid edges from both the intersection and the normal vectors of each face, the width, height, and depth of a cuboid are finally computed as cuboid-shape parameters. Since the parameters can be incrementally updated based on Reference [21], the positional relationships of the planes are analyzed at every frame. This means that both newly detected planes and previously detected cuboids are analyzed so that the false detection of the cuboids can be suppressed with this sequential processing. For example, a falsely detected cuboid face can be replaced with a correct one, and a new plane can be assigned to a previously detected cuboid as a forth cuboid face [23]. In addition, the drift error in the SLAM system is compensated by using the cuboids. Owing to this drift compensation, the duplicated cuboids caused by the fusion failure that occurred in Reference [23] can be reduced. As an application of our framework, we developed an interactive cuboid modeling system to allow users to reconstruct cuboids with augmented reality (AR)-based affordance. In the evaluation, the accuracy of our framework was quantitatively evaluated by using several boxes with their ground truth sizes. The comparison between a batch-based cuboid detection method and our incremental one was also investigated to show the effectiveness of our approach in a cluttered environment. In addition, the accuracy of the cuboid modeling with drift compensation was investigated. Finally, computational cost was explained to show that our framework can run in real time in a room-scale environment.

In summary, the contributions of our paper are listed as follows:

- Cuboid reconstruction is performed by searching three perpendicular planes and computing the intersection of the planes.
- A novel framework for incremental cuboid modeling based on cuboid detection and mapping is proposed.
- The drift error of the SLAM is accurately compensated by using cuboids.
- An application for AR-based interactive cuboid modeling is presented.

This paper is the extended version of Reference [23], and the main difference is twofold: additional process of the drift compensation with the cuboid matching, and its performance for accurate cuboid detection. Due to the drift compensation process, the accumulated error was further corrected, and the accuracy of the cuboid map was improved, as explained in Section 8.3.

## 2. Related Work

Cuboid detection has been investigated in the field of semantic 3D-scene understanding. In this section, we review the literature in terms of the devices used for the detection.

Recognizing cuboids from a single RGB image has been proposed [2,3,4,5,6,24]. Hedau et al. reconstructed a cuboid-based room layout by using vanishing points [2,3]. First, wall, ceiling, and furniture contours were extracted from an input image, and then vanishing points were estimated from three orthogonal straight lines. Finally, a bounding box was aligned to a rectangular area to recognize a cuboid object. Del et al. proposed to use the Manhattan world property such that many surfaces in a room were parallel to three principle ones [4]. This assumption is valid only when cuboids are placed on a floor and parallel to walls. Xiao et al. proposed to first detect vertices on a cuboid based on histograms of oriented gradients, and then detect a cuboid by finding connected edges [5]. Hejrati and Ramanan investigated the performance of several feature representations for categorizing cuboid objects [6]. Dwibedi et al. proposed a deep-learning-based region proposal method for cuboid detection [24]. Basically, cuboid detection using a RGB image is an ill-posed problem, and accuracy is largely degraded under occlusions.

A point cloud acquired from RGB-D images or LIDAR has also been used for cuboid detection [7,8,9,10,11,12,13,14,25,26,27]. Shape descriptors for arbitrary 3D objects were proposed for object classification, including cuboids [7,8,12]. For indoor environments, prior knowledge of a room layout was incorporated to globally optimize object arrangement including cuboids in the room [9,12,27]. To detect buildings as cuboids, a closed polyhedral model is searched from planes detected in a point cloud [25]. An optimization-based approach was proposed by designing a cost function with surfaces, volumes, and their layout to detect cuboids in an RGB-D image [10,11,13,26]. Compared with the approach using a RGB image, the one using a point cloud can provide the metric size and pose of a cuboid in a scene. However, it still suffers from the false detection of cuboids in the presence of sensor noises and registration error. To solve this problem and improve the stability and accuracy of cuboid detection, we propose an incremental approach by fusing multiple measurements captured from different viewpoints without using any constraint on object arrangement.

## 3. Overview

We start by explaining the main steps of our algorithm. First, a plane map, which is composed of oriented planes, is incrementally created from point clouds, and used as the input to our framework. This incremental mapping process is based on our previous method [21] that applies a shape-detection method [28] to incoming point clouds acquired from an RGB-D SLAM system [29]. In other words, any SLAM system can be used as an external tracker, and incrementally produces a noisy 3D point cloud. From such a point cloud, plane are stably detected, and are used for our stable cuboid modeling.

**Figure 1:**
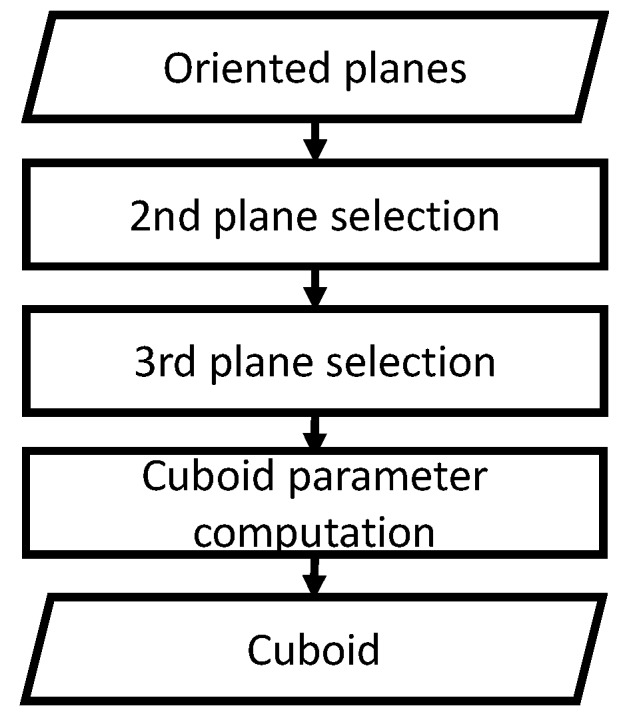
Cuboid detection. From oriented planes, two planes are first selected as a plane pair by checking their positional relationships. Then, the third plane perpendicular to the pair is searched by using the cross product of the two plane normal vectors. Finally, the cuboid-shape parameters are computed from these three planes.

From the plane map, a cuboid map, which is composed of cuboids with their positions, poses, and sizes, is created. As described in Section 4, cuboid faces are detected among a group of planes by analyzing the positional relationships of the planes, namely cuboid check. A cuboid is a convex polyhedron comprising six quadrilateral faces. Also, the adjacent faces of a cuboid are perpendicularly connected. By analyzing these relationships, cuboids can be detected. The procedure of the cuboid detection is illustrated in Figure 1. First, the orthogonality of all the pairs of two planes in the plane map is investigated by brute-force searching. Next, a pair of two perpendicular planes is selected to search their third plane by using the cross product of the two plane normal vectors. Finally, the proximity between the planes is checked. When a set of these three planes passes the cuboid check, the planes are classified as composing a cuboid. By computing the intersection of the planes, the position, pose, and size of a cuboid are determined as cuboid-shape parameters. These parameters can be used in CAD systems.

**Figure 2:**
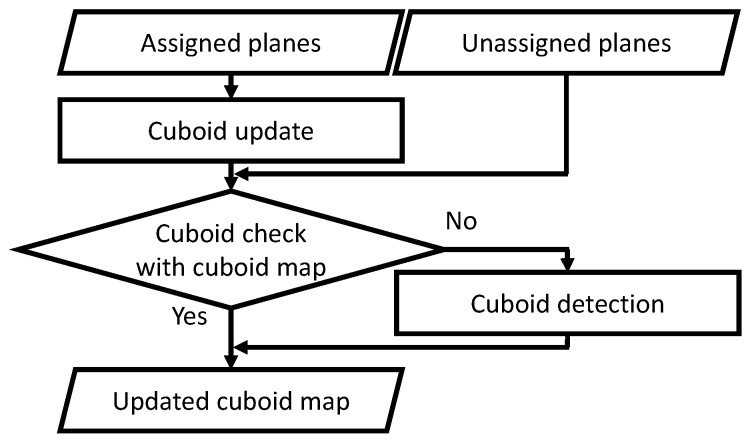
Cuboid mapping. The status of the planes in the plane map is classified into planes assigned to cuboids or not. For the assigned planes, the cuboid check is performed for the cuboids in the map at every frame, as a cuboid update. For the unassigned planes, the cuboid check with the cuboids in the map is first performed, and then cuboid detection is performed if necessary.

To create an accurate cuboid map, an incremental reconstruction process is proposed [23], as described in Section 5. At every frame, the planes in the plane map are classified into planes assigned to cuboids and unassigned ones, as illustrated in Figure 2. First, the cuboid check is performed for the cuboids in the cuboid map to check the positional relationships of the cuboid faces at every frame because their parameters are incrementally updated with [21], This process is specifically referred to as cuboid update. For the unassigned planes, the cuboid check with the cuboid faces in the cuboid map is performed so that the faces in the cuboid map can be replaced with new planes or an undetected cuboid face such as a fourth plane can be assigned to a cuboid in the cuboid map. Then, cuboid detection is performed for the remaining unassigned planes. This incremental process allows users to make the cuboid modeling succeed with AR-based affordance.

**Figure 3:**
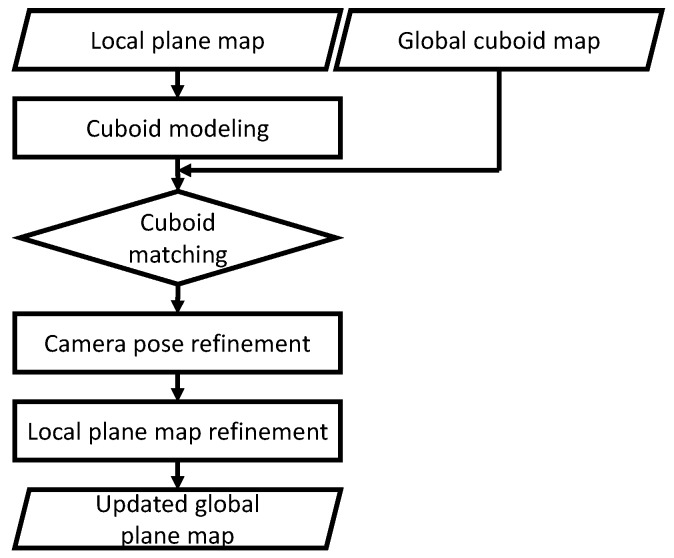
Drift compensation. First, a local plane map is generated with a point cloud from the simultaneous localization and mapping (SLAM), and a cuboid is detected in the local map. Next, the detected cuboid is matched with the one in the global cuboid map in cuboid matching. Then, the transformation from the cuboid in the local map to the one in the global map is computed as the camera pose refinement. Finally, the local map can be merged into the global one with the transformation, as local plane map refinement.

In addition, the drift-compensation process is proposed as described in Section 6. Generally, the map from the SLAM is not accurate when the camera trajectory is long. A loop-closing technique is applied to suppress the error when the closure occurs [30]. However, the error cannot be perfectly suppressed, such that the map may still contain the duplicate of the same object because of the registration error. Therefore, we propose to further suppress the remaining drift error by using the cuboids as illustrated in Figure 3. This process can be considered as the suppression of the registration error or the refinement of the map by using the cuboid. First, a local cuboid map is generated with the point cloud acquired from the SLAM system, and the global map is generated as the collection of the local maps. When a new cuboid is detected in the local map, it is matched with one of the cuboids in the global map to merge the local map into the global one. If the match is detected, the transformation from the local map to the global map can be computed by using the matched cuboids. Otherwise, the local map grows until another cuboid is detected.

## 4. Cuboid Detection

Next, we explain in detail how to detect a cuboid from planes. The first process is to select two perpendicular planes by analyzing the positional relationships. The second process is to search the third plane by using the cross product of the two plane normal vectors. After three perpendicular faces are determined, cuboid-shape parameters are finally determined by computing the intersection of the three planes as cuboid detection.

### 4.1. Second Plane Selection

In this process, sets of two planes composing a cuboid are searched in a brute-force manner. An *i*-th plane in the plane map is parameterized with the center of mass $p_{i}$ and normal vector $n_{i}$ [21]. First, the inner products between a target plane and all of the other planes are computed as an orthogonality check. A plane is selected if the angle computed from the inner product is perpendicular within an error tolerance (e.g., 5 degrees). However, orthogonality is not sufficient for cuboid detection because there are two possibilities of the positional relationship between two planes, concave and convex. A plane can also be selected even if it is far from the target plane and does not compose a cuboid in the environment. Therefore, in the latter processes, these criteria are considered to select an appropriate plane.

**Figure 4:**
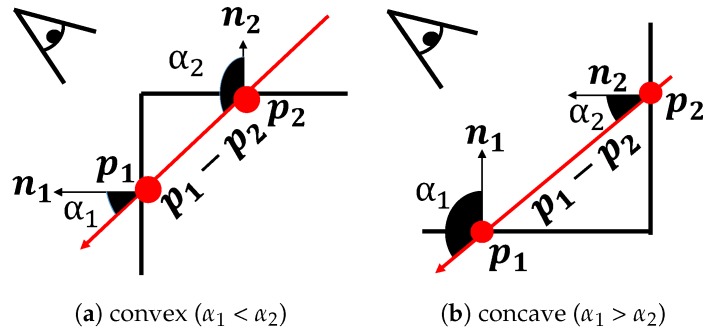
Convexity check. Convexity is analyzed by using the centers of mass and the normal vectors [31].

For the plane selected by the orthogonality check, the convexity with the target plane is analyzed by using Reference [31] as the convexity check. If the relationship between two planes is concave, they do not compose a cuboid because we assume that only outer cuboid faces are visible from a camera. Since each plane has the center of mass and the normal vector, the convexity can be computed from them. In Figure 4, $n_{1}$ and $n_{2}$ are the normal vectors, and $p_{1}$ and $p_{2}$ are the centers, and $\alpha_{1}$ and $\alpha_{2}$ are the angles between a vector $p_{1}-p_{2}$ and each normal vector $n_{1}$ and $n_{2}$, respectively. When $\alpha_{1}$ is smaller than $\alpha_{2}$, the relationship between two planes is convex. Otherwise, the relationship is concave. Therefore, a plane is selected when it satisfies this convex condition: $\alpha_{1}<\alpha_{2}$.

After performing both the orthogonality and convexity checks, there may be multiple candidates for the second plane of a cuboid. In this case, the plane closest to the target plane is finally selected. The distance between each candidate and the target plane is computed by using the center of mass as the proximity check. All of these checks between two planes are referred to as the cuboid check.

### 4.2. Third Plane Selection

From the two perpendicular planes, it is possible to infer a cuboid by using the bounding box for both planes. However, the inference may be incorrect because the 3D edge regions of a cuboid are normally degraded in depth images. To accurately reconstruct a cuboid, three perpendicular planes are used to determine cuboid-shape parameters in our framework.

First, the normalized normal vectors for all of the planes in the plane map are indexed by using a kd-tree, as a plane normal space for fast approximated nearest-neighbor searching in the third plane selection. Then, the cross product of the two perpendicular planes is computed, and is used as a query to the kd-tree to search the third plane of a cuboid. In other words, planes orthogonal to both of the two planes are searched in the space. By using the radius search in the kd-tree with a threshold (e.g., 0.1 for L2 norm between two vectors), the candidates for the third plane are retrieved. Then, the convexity with each of the two planes is checked for each candidate. Finally, the third plane is selected from the candidates according to the proximity check.

### 4.3. Cuboid Parameter Estimation

**Figure 5:**
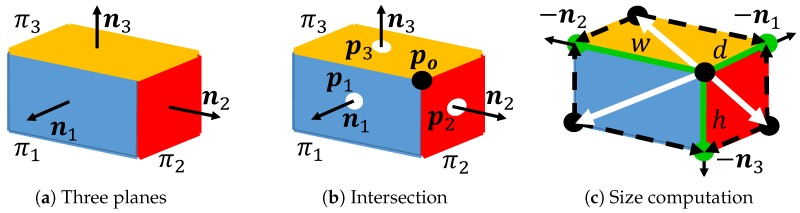
Cuboid-parameter estimation. The intersection of three perpendicular planes is first computed by using the centers of mass and the plane normal vectors. Edges of a cuboid are then determined by using the plane normal vectors. The width, height, and depth are finally computed from the point projection from a plane to an edge.

To reconstruct an accurate cuboid shape, the shape parameters are computed from the three perpendicular planes. In our framework, the parameters are the origin vertex position, three edge directions from the origin, and their lengths. These parameters are useful for standard 3D CAD systems. In Figure 5, $\pi_{i}$ is an *i*-th plane, $n_{i}$ is the normal vector of $\pi_{i}$, and $p_{i}$ is the center of mass, and $p_{o}$ is the intersection of the three perpendicular planes. First, $p_{o}$ is computed by using [22] as follows.

(1) $$\begin{array}{c} p_{o}=\frac{\left(p_{1}\cdot n_{1}\right)\left(n_{2}\times n_{3}\right)}{\left(n_{1}\times n_{2}\right)\cdot n_{3}}+\frac{\left(p_{2}\cdot n_{2}\right)\left(n_{3}\times n_{1}\right)}{\left(n_{1}\times n_{2}\right)\cdot n_{3}}+\frac{\left(p_{3}\cdot n_{3}\right)\left(n_{1}\times n_{2}\right)}{\left(n_{1}\times n_{2}\right)\cdot n_{3}} \end{array}$$

The intersection can be used as the origin of a cuboid to describe cuboid-shape parameters. After determining the intersection, the edge directions from the intersection can automatically be determined because they correspond to the plane normal vectors.

To determine the size of a cuboid, the length of each edge is computed by projecting the points on a plane onto the edge as

(2) $$length=\max_{i}\left\{\left(x_{i}-p_{o}\right)\cdot n\right\}$$

where n is each of the normalized edge-direction vector, and $x_{i}$ is an *i*-th point in the plane. This equation represents that the points on the plane sharing the edge are projected onto the edge, and the furthest point from the intersection on the edge is selected to compute the length. Since each edge is shared by two planes, the average of two lengths is used as a final result. It should be noted that plane number *i* can be arbitrarily determined for the three perpendicular planes.

## 5. Cuboid Mapping

In the plane map, the properties of planes can be divided into two categories, planes assigned to one of the cuboids in the cuboid map, or not. The first category is referred to as assigned planes, and the other is unassigned planes. When a new plane appears in the plane map, it is first considered as an unassigned plane. As illustrated in Figure 2, the process for each plane is different according to the properties. In this section, we explain the details of cuboid mapping.

### 5.1. Cuboid Update

To reduce false detection of cuboids, the cuboid check is performed for all of the cuboid faces in the cuboid map. While capturing only a part of a plane, a cuboid may be wrongly detected as a false positive or a false negative at a time due to the incomplete measurement. Therefore, at every frame, the cuboid check is applied to the cuboids in the map, and cuboid shapes are updated such that some cuboids disappear or others are refined.

This cuboid update is useful for the visual feedback to users of the cuboid modeling system. Normally, users do not understand the best way to capture a scene and when to finish capturing it. By using an incremental approach, the false positives and false negatives of the cuboids can be visualized in an online manner. This largely helps users complete the modeling because they can understand the progress.

As an alternative approach, it is possible to apply a batch-based cuboid-detection method to a point cloud generated from the past at every frame. However, the computational cost at a frame increases according to the size of the point cloud. It is also redundant to detect the cuboids in a point cloud at every frame because the detection result at a frame can be useful at the next frame. In terms of computational efficiency, the incremental approach is appropriate for online systems.

### 5.2. Cuboid Check with Cuboid Map

The unassigned planes in the plain map contain both newly detected planes and previously detected planes that are not assigned to the cuboids. For those planes, the cuboid check with respect to all of the cuboids in the map is first performed.

A cuboid face in the map can be replaced with an unassigned plane when an unassigned plane passes the cuboid check, its normal vector is the same as the cuboid face one, and it is more proximate than the cuboid face. The plane is also assigned to the cuboid if it passes the cuboid check, and it corresponds to a missing face in the cuboid. After this process, the cuboid-shape parameters are updated.

For the remaining unassigned planes, cuboid detection is performed as described in Section 4. After a set of three perpendicular planes is detected, a new cuboid is generated and inserted into the cuboid map.

## 6. Drift Compensation

Any SLAM system cannot avoid the accumulated drift error over time, and always degrades the accuracy of the map. Even though pose graph optimization can reduce the error when the loop is closed [30], the remaining error sometimes still exists. In this section, we explain the process of further suppressing the error with the cuboids.

### 6.1. Overview

**Figure 6:**
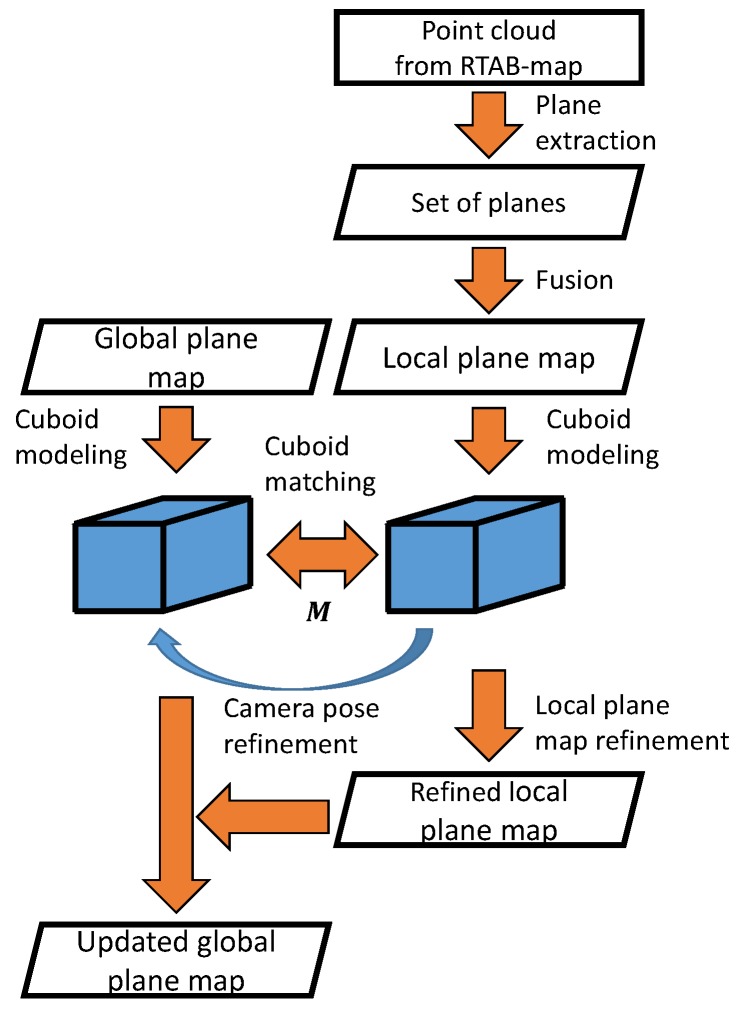
Drift compensation. Cuboids are used to suppress the drift error. First, a local plane map is generated with a point cloud from the SLAM system, and the cuboid is detected in the local map. Then, the detected cuboid is matched with the one in the global map. The transformation matrix is computed by comparing the cuboids in the local map and the global one. Then, the local plane map is refined with the transformation, and is merged into the global one. Once drift compensation is performed, the local plane map is reset.

To create an accurate cuboid map, drift error is compensated by using the cuboids as illustrated in Figure 6. We propose individually using two different maps for planes and cuboids: the local map and the global one. First, the point cloud from the SLAM is used for generating the local plane map. Next, cuboid detection is applied to the local plane map to detect a cuboid. Then, cuboid matching between the local cuboid map and the global one is performed to detect the same cuboid in both the maps. If detected, the local map is merged into the global one by computing the transformation from the cuboid in the local map to the one in the global map. Otherwise, the cuboid remains in the local cuboid map, and another cuboid can be detected after finding additional planes in the local plane map. In our system, we assume that the drift error is small after the pose graph optimization-based map refinement so that cuboid matching can be performed, as implemented in Reference [29]. However, there is still the remaining error. Therefore, we further refine the map by using cuboid-parameter comparison. In other words, our method fails if the overlap does not occur because of the large drift error. It should be noted that both the global plane map and global cuboid one are empty at the beginning of the process. As initialization, the local plane map is directly used as the global one when a cuboid is detected for the first time.

### 6.2. Cuboid Matching

**Figure 7:**
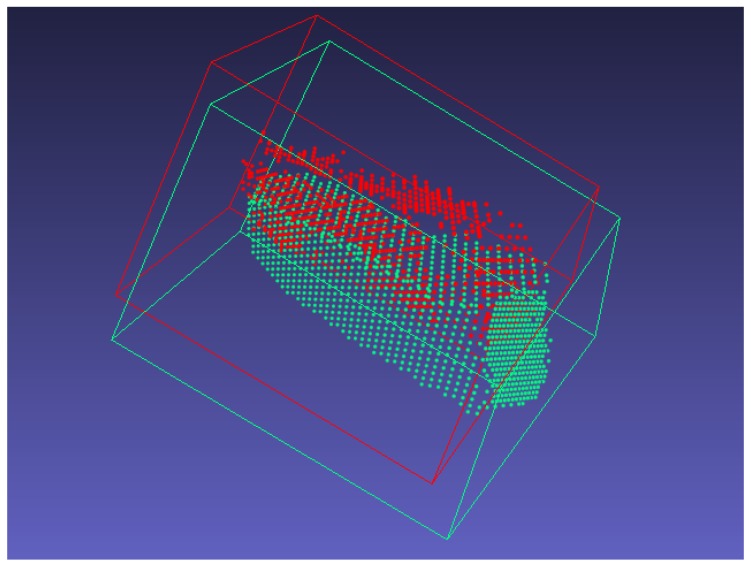
Misalignment between local cuboid (red) and global cuboid (green). Due to the drift error, a duplicate of the same object can arise.

The global map is defined in the world co-ordinate system, and the local map generated with the SLAM system is also the same at the beginning. However, the local map can be gradually shifted due to the accumulation of registration error, and there are duplicates if the local map is simply merged into the global one. To solve this problem, the misalignment of the cuboid is detected by computing the overlapping volume between the axis-aligned bounding box (AABB) of the same cuboid in the local map and global one, as illustrated in Figure 7.

At every frame, cuboid detection is applied in the local map. If a new cuboid is detected, it is checked whether the same cuboid exists in the global map or not. First, the local map is directly merged into the global one. Then, the overlap volume between the AABB of the cuboid in the local map and that in the global one is computed. Finally, the transformation matrix is computed based on the cuboid as described in Section 6.3.

The details of cuboid matching are as follows. The furthest and nearest vertices of the AABB from the origin are detected, and are defined as $C_{f}$ and $C_{n}$, respectively. For instance, the farthest and nearest vertices for the AABB in the global cuboid map are defined as $C_{f_{G}}$ and $C_{n_{G}}$, and those of the local one are defined as $C_{f_{L}}$ and $C_{n_{L}}$. The overlap volume between the AABBs is defined as $V_{O}$, and is computed as follows:

(3) $$\begin{array}{cc} V_{O}= & {\left[\max\left\{0,\min\left\{C_{f_{G}},C_{f_{L}}\right\}-\max\left\{C_{n_{G}},C_{n_{L}}\right\}\right\}\right]}_{xvalue}\\ \times & {\left[\max\left\{0,\min\left\{C_{f_{G}},C_{f_{L}}\right\}-\max\left\{C_{n_{G}},C_{n_{L}}\right\}\right\}\right]}_{yvalue}\\ \times & {\left[\max\left\{0,\min\left\{C_{f_{G}},C_{f_{L}}\right\}-\max\left\{C_{n_{G}},C_{n_{L}}\right\}\right\}\right]}_{zvalue} \end{array}$$

where ${\left[\;\right]}_{ivalue}$ means the value of *i* axis (i=x,y,z). If $V_{O}$ is greater than zero, the cuboid is considered as overlapped.

In cuboid matching, two or more duplicate cuboids may appear. In this case, the cuboid with the highest overlap rate is selected. The volume of the AABB in the global cuboid map is defined as $V_{G}$, and the overlap rate is defined as rate. $V_{G}$ and rate are computed as follows.

(4) $$\begin{array}{ccc} V_{G} & = & {\left[C_{f_{G}}-C_{n_{G}}\right]}_{xvalue}\times {\left[C_{f_{G}}-C_{n_{G}}\right]}_{yvalue}\times {\left[C_{f_{G}}-C_{n_{G}}\right]}_{zvalue} \end{array}$$

(5) $$\begin{array}{ccc} rate & = & V_{O}/V_{G}\; \end{array}$$

### 6.3. Camera Pose Refinement

After cuboid matching, the camera pose of the SLAM in the local map is refined with the duplicate of the same cuboid to be consistent with the global map. The relationship among a point in global map $X_{G}$, a point in the local one $X_{L}$, and transformation matrix M is

(6) $$\begin{array}{ccc} {\tilde{X}}_{G} & = & \left[\begin{array}{cc} R & t\\ 0^{T} & 1 \end{array}\right]{\tilde{X}}_{L} \end{array}$$

(7) $$\begin{array}{ccc} M & = & \left[\begin{array}{cc} R & t\\ 0^{T} & 1 \end{array}\right]\; \end{array}$$

where ${\tilde{X}}_{G}$ and ${\tilde{X}}_{L}$ are homogeneous coordinates, R is a rotation matrix, and t is a translation vector. $P_{G}$ and $P_{L}$ are the pose of the cuboid in global and local map, respectively, and are defined as:

(8) $$P_{G}=\left[\begin{array}{cc} R_{G} & t_{G}\\ 0^{T} & 1 \end{array}\right]$$

(9) $$P_{L}=\left[\begin{array}{cc} R_{L} & t_{L}\\ 0^{T} & 1 \end{array}\right]$$

Cuboid rotation is computed from the normal vectors of three perpendicular planes $n_{i}$, as illustrated in Figure 5. As a notation, the normal of the first plane is $n_{1}$, the second one is $n_{2}$, and the third one is $n_{3}$. Cuboid rotations are formed as follows:

(10) $$\begin{array}{ccc} R_{G} & = & \left[\begin{array}{ccc} n_{1_{G}} & n_{2_{G}} & n_{3_{G}} \end{array}\right] \end{array}$$

(11) $$\begin{array}{ccc} R_{L} & = & \left[\begin{array}{ccc} n_{1_{L}} & n_{2_{L}} & n_{3_{L}} \end{array}\right] \end{array}$$

R and t are, finally, computed as follows.

(12) $$\begin{array}{ccc} R & = & R_{G}{R_{L}}^{T}\; \end{array}$$

(13) $$\begin{array}{ccc} t & = & p_{o_{G}}-Rp_{o_{L}} \end{array}$$

where $p_{o_{G}}$ and $p_{o_{L}}$ are the origin of local and global cuboids, respectively. t is computed by using the origin point of the cuboid.

### 6.4. Local Plane Map Refinement

The local plane map is refined by using M computed in the camera pose refinement. The plane parameters are a normal vector $n_{L}$, and an arbitrary point on plane $V_{L}$. First, $n_{L}$ is rotated, and $V_{L}$ is transformed as

(14) $$\begin{array}{ccc} n_{G} & = & Rn_{L}\; \end{array}$$

(15) $$\begin{array}{ccc} {\tilde{V}}_{G} & = & \left[\begin{array}{cc} R & t\\ 0^{T} & 1 \end{array}\right]{\tilde{V}}_{L} \end{array}$$

where $n_{G}$ is the normal vector of the refined plane, and $V_{G}$ is an arbitrary point on the refined plane. Then, all points on the plane are projected following the point-projection method [21]. Finally, the refined map is merged into the global one.

**Figure 8:**
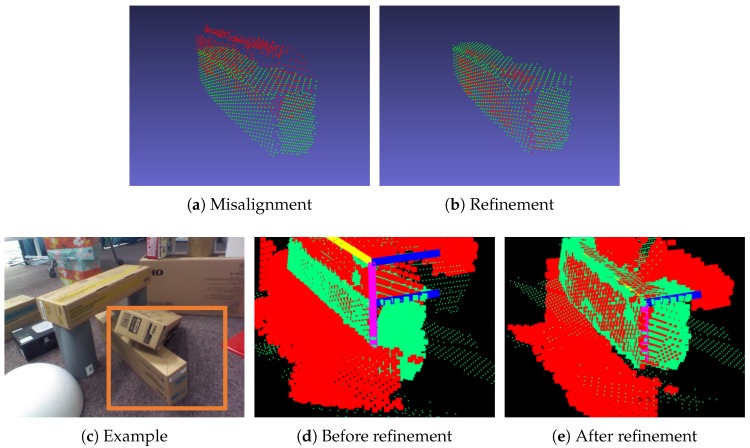
Local plane map refinement. The red and green represent the cuboid in the local and global map, respectively. The cuboid faces are not aligned in (**a**) after directly merging the local plane map into the global one. The pose of a cuboid is refined in (**b**) after camera pose refinement. For example, scenes (**c**,**d**) represent the direct merger of the local map into the global map, and (**e**) is the result of the refinement. The edges of each cuboid are visualized, and there is a gap between the cuboids in (**d**), whereas the cuboids are merged in (**e**).

Figure 8 illustrates the results of this refinement process. The red and green planes represent detected cuboids in the local and global map, respectively. When directly merging the local map with the global one, as illustrated in Figure 8a, a duplicate may occur. After refinement, the duplicate disappears, as illustrated in Figure 8b. Other planes in the local plane map are also transformed. In the area of the orange rectangle in Figure 8c, cuboid matching and camera pose refinement are performed by using one of the cuboids. As illustrated in Figure 8d, there is misalignment before refinement. For instance, the difference between the positions of the local and global cuboid was 5.8 cm in this case. After refinement, many planes are well-aligned, as illustrated in Figure 8e. The misalignment between cuboid faces and other planes is reduced.

## 7. Interactive Cuboid Modeling

Since 3D modeling using a camera is not an easy task for nonexperts, interactive techniques have been proposed [32]. For instance, the result of 3D modeling can easily be modified on user interfaces [33,34]. The incompleteness of the modeling is also visualized by showing a 2D slide of a point cloud for modeling a room [35] or showing an example for modeling an object [36]. Here, we introduce a simple but effective affordance for modeling a cuboid.

**Figure 9:**
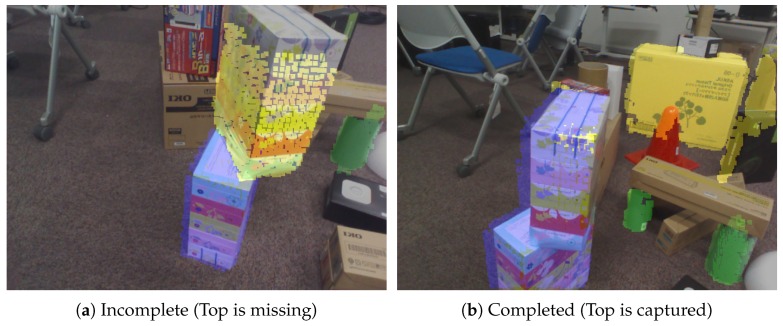
Interactive cuboid modeling. Yellow and blue regions represent incomplete and completed, respectively. A box is represented by yellow in (**a**), and the color becomes blue in (**b**) after the user completely captures it.

As illustrated in Figure 9, the points on planes are overlaid with colors. Blue and yellow regions represent detected cuboids as completed ones and two perpendicular planes as incomplete ones, respectively. In other words, the color represents the modeling progress. To model a cuboid, the user’s task is to find yellow regions and then capture the remaining plane where colored points are not overlaid, as illustrated in Figure 9a. This corresponds to the instruction for the users. Since users are induced to capture the remaining plane from the visualization, this interaction can be regarded as AR-based affordance. After the user successfully captures the cuboid, the color of the cuboid becomes blue, as illustrated in Figure 9b. Owing to the incremental approach, it is possible to develop this type of interactive modeling systems.

## 8. Evaluation

To evaluate the performance of our proposed framework, we first prepared RGB-D image sequences capturing multiple boxes as our dataset because only a dataset for single views was developed in the literature [5] and there is no dataset with RGB-D sequences containing cuboid ground truth annotations. For the camera, a Kinect V1 sensor was used; therefore, the boxes were set up in indoor environments. The size of each box was measured by a ruler as a ground truth.

For the evaluation criterion, the accuracy of each estimated cuboid shape was investigated by comparing the result with its ground truth. To investigate the effectiveness of our proposed method, batch-based cuboid detection in a point cloud was implemented as a benchmarking method, and its result was compared with our result. Finally, the computational cost of each process was measured.

### 8.1. Cuboid-Shape Estimation

**Figure 10:**
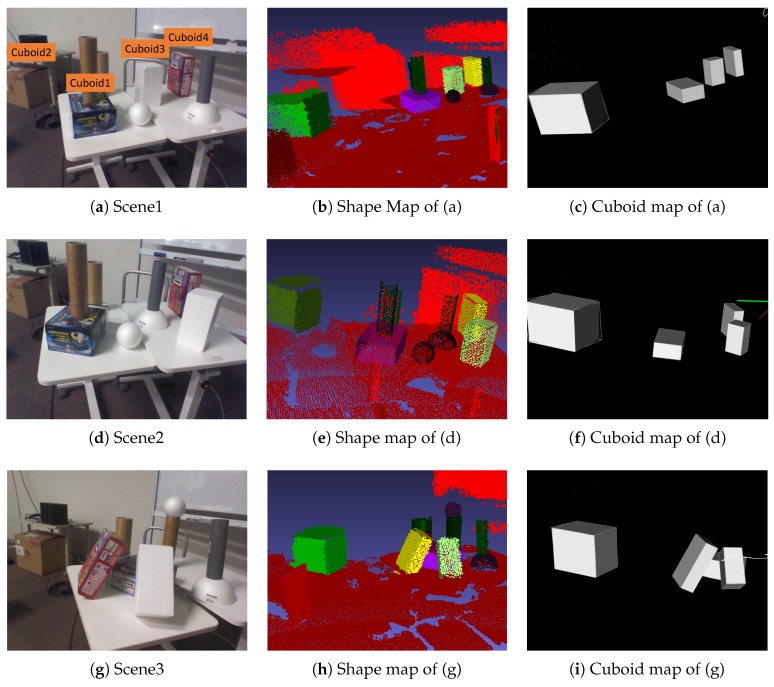
Cuboid-shape estimation at various scenes. Three datasets were designed to investigate the accuracy of estimated cuboid shapes according to the arrangement. The first, the second, and their columns represent a scene, its shape map [21], and its cuboid map, respectively. The size of each cuboid was measured by a ruler as ground truth.

As illustrated in Figure 10, three scenes were designed so that four cuboids with different sizes were arranged on a table and other objects were placed as obstacles. In Scene 1, the cuboids were rotated to face the same direction. In Scene 2, the cuboids were rotated to face in different directions except for the top face. In Scene 3, two cuboids were inclined onto a cuboid. In all scenes, one cuboid was placed far from the table. In Figure 10, the first column represents an example image of each scene, the second represents the shape map [21] drawn with different colors per cuboid, and the third represents our cuboid map. At each scene, an RGB-D image sequence was freely captured from one side of the table by moving around the table.

In this experiment, all cuboids were successfully detected regardless of the cuboid arrangements with some occlusions, and their shape parameters were also computed. The estimated size of each cuboid at each scene is described in Table 1. The error of Cuboid 2 was larger than the others because this cuboid was located at the farthest position from the table. This resulted from the accuracy degradation of depth images. The shape of Cuboid 2 was not completely measured because an obstacle hid it. In this case, the accuracy was largely decreased. For other cuboids, error variance was relatively small.

### 8.2. Cuboid Detection in a Cluttered Environment

To show the effectiveness of our incremental approach, a batch-based approach was implemented as follows. An RGB-D SLAM system [29] was applied to an RGB-D image sequence to generate a full point cloud in a scene. Next, a shape-detection method [28] was applied to the point cloud to detect planes in the scene. Since each plane normal vector cannot be uniquely determined, two planes having opposite normal vectors were generated from one plane. Then, the cuboid detection in Section 4 was applied to all the oriented planes to detect cuboids.

**Figure 11:**
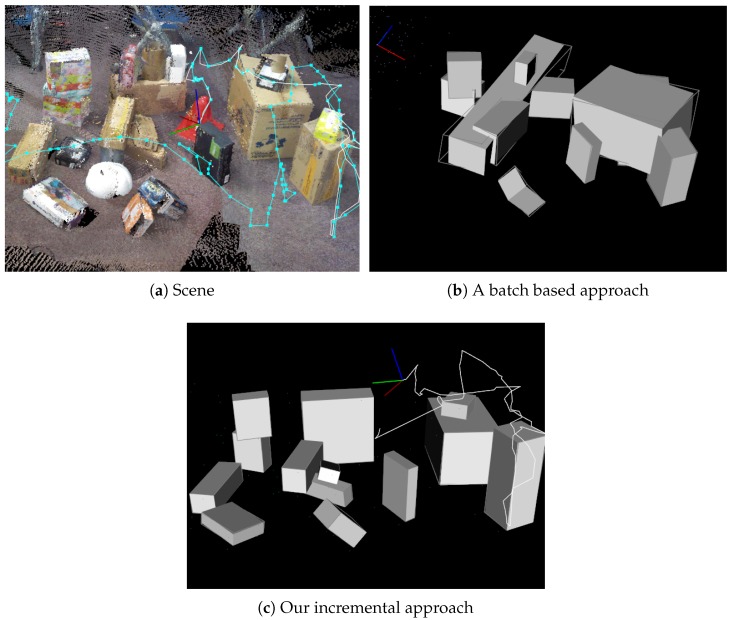
Comparison between a batch-based approach with our incremental one. For (**a**) a scene reconstructed by Reference [29], we applied batch-based cuboid detection to the scene, and had (**b**) as the result. Comparing (**c**) with our result, there were many false positives and false negatives because of noisy point cloud reconstruction. Accuracy details are presented in Table 2.

For this experiment, a challenging scene was designed as illustrated in Figure 11. In this scene, 19 cuboids were randomly arranged, and many other objects were placed as a cluttered environment. The scene was captured by freely moving around the scene. It should be noted that our visual guidance system was not used to capture the dataset.

**Figure 12:**
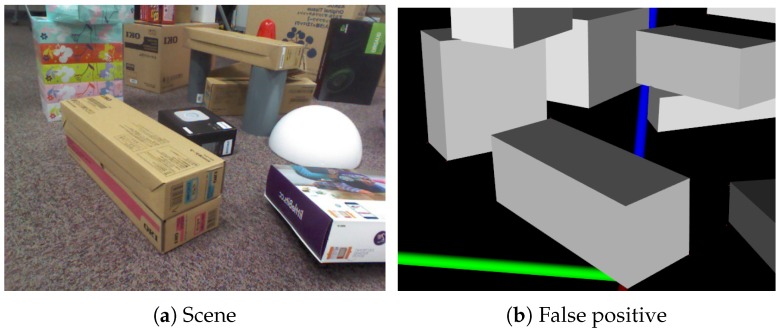
An example of a false-positive result. In (**a**), a box is stacked on another with the same size. It is difficult to distinguish the boxes in (**b**).

The performance of the method was evaluated in terms of precision and recall based on false positives, false negatives, and true positives, as described in Table 2. The results of the cuboid maps are illustrated in Figure 11. In the batch-based approach, there were more false positives and false negatives compared with our approach. Since the point cloud from RGB-D SLAM was noisy due to the registration error, several false-positive and false-negative planes were detected. The ambiguity of plane normals also caused the wrong clustering of three perpendicular planes. In our approach, cuboids were correctly detected because most of the planes were accurately modeled by avoiding the influence of error accumulation in Iterative Closest Point (ICP)-based D-SLAM [21]. However, false negatives still occurred in our approach due to the incomplete measurement of the cuboids. For instance, it was still difficult to detect two stacked boxes with similar sizes when they were aligned, as illustrated in Figure 12. We discuss this in detail as a limitation in Section 8.5.

### 8.3. Effectiveness of Drift Compensation

**Figure 13:**
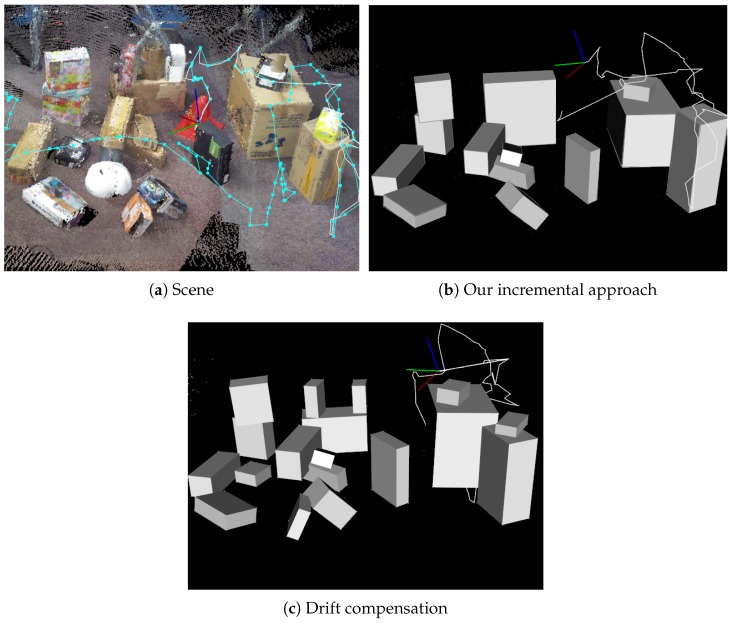
Effectiveness of drift compensation. For (**a**) a scene reconstructed by Reference [29], we applied our incremental cuboid detection to the scene, and (**b**) was the result. Comparing (**c**) with drift compensation, accuracy details are presented in Table 2.

The performance of cuboid detection with drift compensation was evaluated in terms of precision and recall, the same as Section 8.2. The results are described in Table 3. The results of the cuboid maps are also illustrated in Figure 13. In our incremental approach without drift compensation, five cuboids were missing. This is because cuboid faces cannot be properly detected when camera pose estimation error became large and the local map receded from the global one. By incorporating drift compensation, the number of detected cuboids increased owing to camera pose refinement.

### 8.4. Computational Cost

**Figure 14:**
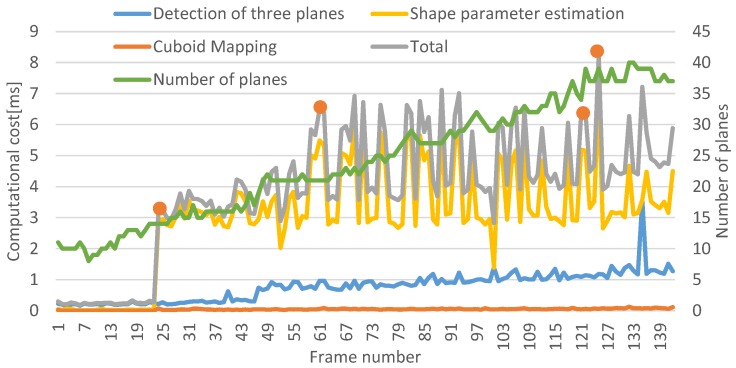
Computational cost. Orange dots represent the time when a new cuboid is detected. The cost of shape-parameter estimation was larger than others. The detection of three planes represents the sum of both second and third plane detections in Section 4. This cost increased as time passed because the number of unassigned planes increased.

The computational cost was measured at Scene 1 in Figure 10a with 3.70 GHz of Intel (R) Xeon (R) CPU E 5-1620 v2, as illustrated in Figure 14. In Reference [21], the computational cost required for RGB-D SLAM and plane reconstruction was within 100 ms, on average. Compared to Reference [21], we focused on measuring the cost for 3D cuboid modeling. In cuboid detection, the costs of detecting three planes and computing shape parameters were separately measured. In the figure, orange dots represent the time when a new cuboid is detected.

The shape parameter estimation needed most computational costs, especially in the process of point projection to a line to compute edge lengths. The cost of detecting three planes gradually increased according to the number of planes in the map because planes can be detected not only from cuboids but also noncuboids such as walls. In this case, the cuboid check was applied to the planes from noncuboids in every frame. Therefore, this process affected the cost increase. Overall, the cost of our framework in a room-scale environment was sufficient for running with RGB-D SLAM.

### 8.5. Limitation

**Figure 15:**
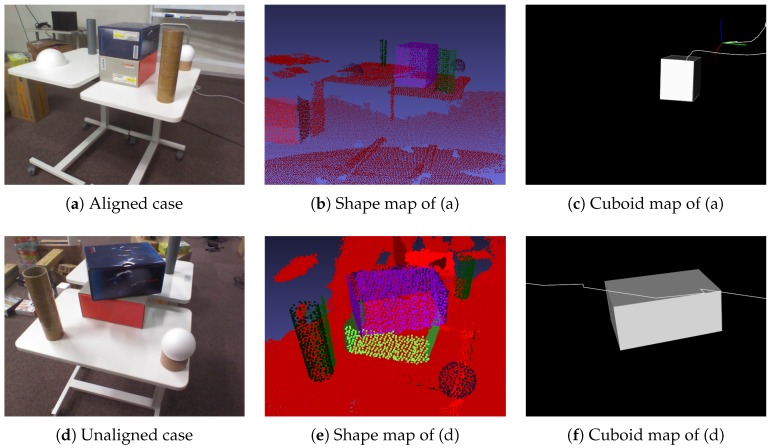
Limitation. In the first row, two stacked boxes are detected as one cuboid when they are aligned. In the second row, even when two stacked boxes are not aligned, the lower box is not detected because the top face of the lower box is not sufficiently captured as a plane. The accuracy of cuboid detection can be degraded when boxes are stacked according to the arrangement.

As illustrated in Figure 15, cuboid detection using three perpendicular planes sometimes failed when boxes were stacked. Basically, detection accuracy depends on the quality of the plane map. For instance, two stacked boxes can be detected as one cuboid when they are aligned. Since the faces of the two boxes compose a plane, they are detected as one plane. By using image-based segmentation, two boxes can be separately detected. In another case, the lower box cannot be detected even when two stacked boxes are not aligned because the top face of the lower box is still hidden by the upper box. Since three perpendicular planes are required to detect a cuboid in our framework, detection fails.

## 9. Conclusions

We presented a framework for generating a cuboid map in an incremental manner. In this approach, a cuboid is first detected by analyzing the positional relationship between oriented planes. Then, it is incrementally updated to suppress false detections. In addition, the drift-compensation process is proposed to create the consistent and accurate cuboid map. False detection is further suppressed by using the removed duplicate plane map. An interactive cuboid-modeling system was designed to assist users in reconstructing cuboids.

This evaluation demonstrated that cuboid modeling with our approach is more accurate than a batch-based method. In addition, the evaluation demonstrated that cuboid modeling with the drift-compensation process was more accurate than without drift compensation. Our method also successfully detected cuboids regardless of their arrangements. However, three perpendicular planes are required to be captured to compute cuboid-shape parameters, as our limitation.

In future work, the performance of our framework with various additional scenes will be investigated. Image features from RGB images will also be integrated into our framework to increase the accuracy and robustness of cuboid detection, since the point cloud is obtained by RTAB-MAP [29], which is relatively inaccurate in terms of reconstruction quality compared with a TSDF based fusion method such as KinectFusion [37]. 

## Figures and Tables

**Table 1:** Estimated cuboid lengths (cm).

**Cuboid 1**	**Depth**	**Width**	**Height**	**Cuboid 2**	**Depth**	**Width**	**Height**
*Ground truth*	16.0	22.5	10.5	*Ground truth*	46.6	49.8	41.0
*Scene 1*	16.8	23.5	9.8	*Scene1*	46.9	50.3	34.1
*Scene 2*	16.8	23.1	9.6	*Scene2*	40.9	50.7	36.5
*Scene 3*	16.3	24.6	9.8	*Scene3*	39.8	47.1	31.2
**Cuboid 3**	**Depth**	**Width**	**Height**	**Cuboid 4**	**Depth**	**Width**	**Height**
*Ground truth*	9.8	9.8	19.7	*Ground truth*	7.9	16.0	21.8
*Scene 1*	10.3	10.7	19.2	*Scene 1*	7.0	17.2	21.3
*Scene 2*	10.7	11.4	19.4	*Scene 2*	7.4	19.4	21.9
*Scene 3*	9.9	10.5	19.3	*Scene 3*	7.2	16.7	21.2

**Table 2:** Performance of cuboid detection.

	Batch	Ours
*Precision*	0.64	0.92
*Recall*	0.37	0.63
**Batch**	*Positive*	*Negative*
*True*	7	-
*False*	4	12
**Ours**	*Positive*	*Negative*
*True*	12	-
*False*	1	7

**Table 3:** Performance of cuboid detection.

	Incremental	Drift Compensation
*Precision*	0.92	0.94
*Recall*	0.63	0.89
**Incremental**	*Positive*	*Negative*
*True*	12	-
*False*	1	7
**Drift compensation**	*Positive*	*Negative*
*True*	17	-
*False*	1	2

## References

[1] Saxena A., Driemeyer J., Ng A.Y. (2008). Robotic grasping of novel objects using vision. Int. J. Robot. Res..

[2] Hedau V., Hoiem D., Forsyth D. Recovering the spatial layout of cluttered rooms. Proceedings of the IEEE 12th International Conference on Computer Vision.

[3] Hedau V., Hoiem D., Forsyth D. (2010). Thinking inside the box: Using appearance models and context based on room geometry. European Conference on Computer Vision.

[4] Del Pero L., Guan J., Brau E., Schlecht J., Barnard K. Sampling bedrooms. Proceedings of the IEEE Conference on Computer Vision and Pattern Recognition (CVPR).

[5] Xiao J., Russell B., Torralba A. (2012). Localizing 3D cuboids in single-view images. Advances in Neural Information Processing Systems.

[6] Hejrati M., Ramanan D. Categorizing cubes: Revisiting pose normalization. Proceedings of the IEEE Winter Conference on Applications of Computer Vision (WACV).

[7] Osada R., Funkhouser T., Chazelle B., Dobkin D. (2002). Shape distributions. ACM Trans. Graph. (TOG).

[8] Rusu R.B., Marton Z.C., Blodow N., Dolha M., Beetz M. (2008). Towards 3D point cloud based object maps for household environments. Robot. Auton. Syst..

[9] Lin D., Fidler S., Urtasun R. Holistic scene understanding for 3d object detection with rgbd cameras. Proceedings of the IEEE International Conference on Computer Vision (ICCV).

[10] Jiang H., Xiao J. A linear approach to matching cuboids in RGBD images. Proceedings of the IEEE Conference on Computer Vision and Pattern Recognition (CVPR).

[11] Khan S.H., He X., Bannamoun M., Sohel F., Togneri R. Separating objects and clutter in indoor scenes. Proceedings of the IEEE Conference on Computer Vision and Pattern Recognition (CVPR).

[12] Ren Z., Sudderth E.B. Three-dimensional object detection and layout prediction using clouds of oriented gradients. Proceedings of the IEEE Conference on Computer Vision and Pattern Recognition.

[13] Hashemifar Z.S., Lee K.W., Napp N., Dantu K. Consistent Cuboid Detection for Semantic Mapping. Proceedings of the IEEE 11th International Conference on Semantic Computing (ICSC).

[14] Nguyen T., Reitmayr G., Schmalstieg D. (2015). Structural modeling from depth images. IEEE Trans. Vis. Comput. Graph..

[15] Balsa-Barreiro J., Fritsch D. (2015). Generation of 3D/4D photorealistic building models. The testbed area for 4D Cultural Heritage World Project: The historical center of Calw (Germany). International Symposium on Visual Computing.

[16] Balsa-Barreiro J., Fritsch D. (2018). Generation of visually aesthetic and detailed 3D models of historical cities by using laser scanning and digital photogrammetry. Digit. Appl. Archaeol. Cult. Herit..

[17] Reutebuch S.E., Andersen H.E., McGaughey R.J. (2005). Light detection and ranging (LIDAR): An emerging tool for multiple resource inventory. J. Forest..

[18] Roberto R.A., Uchiyama H., Lima J.P.S., Nagahara H., Taniguchi R.I., Teichrieb V. (2017). Incremental structural modeling on sparse visual SLAM. IPSJ Trans. Comput. Vis. Appl..

[19] Roberto R., Lima J.P., Uchiyama H., Arth C., Teichrieb V., Taniguchi R., Schmalstieg D. Incremental Structural Modeling Based on Geometric and Statistical Analyses. Proceedings of the IEEE Winter Conference on Applications of Computer Vision (WACV).

[20] Pradeep V., Rhemann C., Izadi S., Zach C., Bleyer M., Bathiche S. MonoFusion: Real-time 3D reconstruction of small scenes with a single web camera. Proceedings of the IEEE International Symposium on Mixed and Augmented Reality (ISMAR).

[21] Olivier N., Uchiyama H., Mishima M., Thomas D., Taniguchi R., Roberto R., Lima J.P., Teichrieb V. Live Structural Modeling using RGB-D SLAM. Proceedings of the 2018 IEEE International Conference on Robotics and Automation (ICRA).

[22] Goldman R. (1990). Intersection of three planes. Graphics Gems.

[23] Mishima M., Uchiyama H., Thomas D., Taniguchi R., Roberto R., Lima J.P., Teichrieb V. (2018). RGB-D SLAM based Incremental Cuboid Modeling. http://www.sys.info.hiroshima-cu.ac.jp/3drw2018/procs/W17-08.pdf.

[24] Dwibedi D., Malisiewicz T., Badrinarayanan V., Rabinovich A. (2016). Deep cuboid detection: Beyond 2d bounding boxes. arXiv.

[25] Nguatem W., Drauschke M., Mayer H. (2012). Finding cuboid-based building models in point clouds. ISPRS Int. Arch. Photogramm. Remote Sens. Spat. Inf. Sci..

[26] Zhang C., Hu Y. (2017). CuFusion: Accurate real-time camera tracking and volumetric scene reconstruction with a cuboid. Sensors.

[27] Rodriguez-Garavito C., Camacho-Munoz G., Álvarez-Martínez D., Cardenas K.V., Rojas D.M., Grimaldos A. (2018). 3D object pose estimation for robotic packing applications. WEA 2018: Applied Computer Sciences in Engineering.

[28] Schnabel R., Wahl R., Klein R. (2007). Efficient RANSAC for point-cloud shape detection. Computer Graphics Forum.

[29] Labbé M., Michaud F. Online global loop closure detection for large-scale multi-session graph-based slam. Proceedings of the IEEE/RSJ International Conference on Intelligent Robots and Systems (IROS 2014).

[30] Labbe M., Michaud F. (2013). Appearance-based loop closure detection for online large-scale and long-term operation. IEEE Trans. Robot..

[31] Stein S.C., Wörgötter F., Schoeler M., Papon J., Kulvicius T. Convexity based object partitioning for robot applications. Proceedings of the IEEE International Conference on Robotics and Automation (ICRA).

[32] Sinha S.N., Steedly D., Szeliski R., Agrawala M., Pollefeys M. (2008). Interactive 3D architectural modeling from unordered photo collections. ACM Trans. Graph. (TOG).

[33] Shao T., Xu W., Zhou K., Wang J., Li D., Guo B. (2012). An interactive approach to semantic modeling of indoor scenes with an rgbd camera. ACM Trans. Graph. (TOG).

[34] Zhang Y., Luo C., Liu J. (2012). Walk&sketch: Create floor plans with an rgb-d camera. Proceedings of the ACM Conference on Ubiquitous Computing.

[35] Du H., Henry P., Ren X., Cheng M., Goldman D.B., Seitz S.M., Fox D. Interactive 3D modeling of indoor environments with a consumer depth camera. Proceedings of the 13th International Conference on Ubiquitous Computing.

[36] Kim Y.M., Mitra N.J., Huang Q., Guibas L. (2013). Guided Real-Time Scanning of Indoor Objects. Computer Graphics Forum.

[37] Newcombe R.A., Izadi S., Hilliges O., Molyneaux D., Kim D., Davison A.J., Kohi P., Shotton J., Hodges S., Fitzgibbon A. KinectFusion: Real-time dense surface mapping and tracking. Proceedings of the 10th IEEE International Symposium on Mixed and Augmented Reality (ISMAR).

